# Untargeted Metabolomics and Quantitative Analysis of Tryptophan Metabolites in Myalgic Encephalomyelitis Patients and Healthy Volunteers: A Comparative Study Using High-Resolution Mass Spectrometry

**DOI:** 10.1021/acschemneuro.4c00444

**Published:** 2024-09-20

**Authors:** Sandy Abujrais, Theodosia Vallianatou, Jonas Bergquist

**Affiliations:** † Analytical Chemistry and Neurochemistry, Department of Chemistry—BMC, Uppsala University, Box 599, 751 24 Uppsala, Sweden; ‡ The ME/CFS Collaborative Research Centre at Uppsala University, 751 24 Uppsala, Sweden; § Spatial Mass Spectrometry, Department of Pharmaceutical Biosciences, Uppsala University, Box 591, 751 24 Uppsala, Sweden

**Keywords:** high resolution mass spectrometry, myalgic encephalomyelitis (ME/CFS), neurodegenerative disorders, metabolomics, targeted analysis, untargeted analysis, biomarker

## Abstract

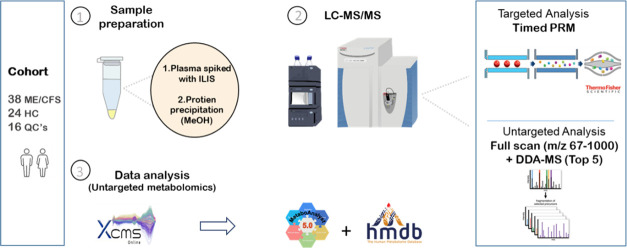

Myalgic encephalomyelitis/chronic fatigue syndrome (ME/CFS) is a chronic, complex illness characterized by severe and often disabling physical and mental fatigue. So far, scientists have not been able to fully pinpoint the biological cause of the illness and yet it affects millions of people worldwide. To gain a better understanding of ME/CFS, we compared the metabolic networks in the plasma of 38 ME/CFS patients to those of 24 healthy control participants. This involved an untargeted metabolomics approach in addition to the measurement of targeted substances including tryptophan and its metabolites, as well as tyrosine, phenylalanine, B vitamins, and hypoxanthine using liquid chromatography coupled to mass spectrometry. We observed significant alterations in several metabolic pathways, including the vitamin B3, arginine-proline, and aspartate-asparagine pathways, in the untargeted analysis. The targeted analysis revealed changes in the levels of 3-hydroxyanthranilic acid, 3-hydroxykynurenine, hypoxanthine, and phenylalanine in ME/CFS patients compared to the control group. These findings suggest potential alterations in immune system response and oxidative stress in ME/CFS patients.

## 1 Introduction

Myalgic encephalomyelitis/chronic fatigue syndrome (ME/CFS) is a debilitating multisystem disorder. Symptoms of the condition range from mild to severe, with the most severely affected individuals enduring ongoing fatigue, frequent pain, and postexertional malaise (PEM), which is the exacerbation and onset of new symptoms after exertion, both physical and mental. Additionally, these individuals often experience heightened sensitivity to light, touch, sound, smell, certain foods, and medications.^1^ ME/CFS is estimated to affect between 17 and 24 million people worldwide.^2^ Determining the exact prevalence of the condition is challenging due to factors such as underdiagnosis and misdiagnosis.^3^ Despite its significant impact, ME/CFS remains one of the most poorly studied medical conditions. Although its biological basis has remained elusive, emerging evidence suggests that ME/CFS involves multiorgan perturbations across the immune, metabolic, and neuroendocrine systems.^4^

Studies into the metabolic profile of ME/CFS patients have suggested a potential link between altered metabolism and the pathogenesis of ME/CFS.^5^ In recent years, untargeted metabolomics has emerged as a powerful tool to provide a comprehensive characterization of a biological sample’s metabolome without prior selection of the compounds, via techniques such as liquid chromatography coupled to mass spectrometry (LC-MS) or nuclear magnetic resonance (NMR) analysis. For instance, untargeted analysis using an NMR method in ME/CFS patients revealed increased glucose and decreased levels of acetate, glutamate, hypoxanthine, lactate, and phenylalanine in serum.^6^ These changes suggest inhibited glycolysis and oxidative stress pathways, as well as reduced amino acid levels. Another study, using an untargeted metabolomics via LC-MS/MS method, identified disruptions in four metabolic pathways: cofactors and vitamins, energy, nucleotides, and peptides. It also highlighted heme redox imbalance and alterations in α-ketoglutarate related to the tricarboxylic acid (TCA) cycle.^7^

Among the various metabolic pathways that have the potential to play a role in ME/CFS, the tryptophan metabolic pathway has gained attention due to its involvement in immune function, neurotransmission and energy metabolism.^8^ In terms of immune function, tryptophan degradation by enzymes like indoleamine 2,3-dioxygenase (IDO1) and tryptophan 2,3-dioxygenase (TDO2) produces metabolites such as kynurenine, which have been shown to drive immunosuppressive actions in various cancers.^9^ These metabolites can inhibit the activity of immune cells, particularly T cells and suppress the immune responses.^9^ Regarding neurotransmission, a smaller portion of tryptophan is metabolized toward the serotonin (5-hydroxytryptamine, 5-HT) pathway, which subsequently converts to melatonin, a regulator of the sleep-wake cycle.^8^ Alterations in tryptophan metabolism can thus impact neurotransmitter levels, particularly 5-HT, which is crucial for mood regulation.^8^ Moreover, tryptophan plays a pivotal role in energy production through the kynurenine pathway, leading to the synthesis of NAD+, an essential molecule for cellular energy metabolism.^8^

Discrepancies in the literature regarding the kynurenine pathway activation in ME/CFS highlight the challenges in comparing findings due to different sample types and analytical methods. For example, one study utilized liquid chromatography with an ultraviolet detector (LC-UV) and gas chromatography coupled to mass spectrometry (GC-MS) to identify persistent activation of the kynurenine pathway in the plasma of ME/CFS patients. This activation was marked by increased production of kynurenine and an elevated kynurenine/tryptophan ratio, alongside decreased levels of 3-hydroxykynurenine, kynurenic acid, picolinic acid, and quinolinic acid compared to healthy controls.^10^ Conversely, another study used LC-UV and LC coupled to mass spectrometry (LC-MS) to observe lower kynurenine and higher 3-hydroxykynurenine levels in the serum of ME/CFS patients compared to healthy controls, with no significant differences observed in quinolinic or kynurenic acids. These discrepancies emphasize the need for standardized inclusion criteria for ME/CFS patients and methodologies in the analysis of samples e.g., tryptophan metabolites to achieve more consistent and comparable results.^5^

This study uses liquid chromatography coupled with high-resolution mass spectrometry (LC-HRMS) for untargeted metabolomics to identify broad metabolic changes in plasma samples from ME/CFS patients and healthy controls. Significant disruptions in multiple pathways were identified. We then analyzed tryptophan metabolites to identify specific alterations in ME/CFS patients. Integrating untargeted and targeted metabolomics, aims to comprehensively assess metabolic changes in ME/CFS plasma, offering both broad insights and detailed characterization of dysregulations to enhance understanding of ME/CFS pathophysiology.

## 2 Results and Discussion

### 2.1 Overview of the Data with Untargeted Analysis

The results obtained from XCMS online revealed a total of 3543 features in positive ionization mode after going through the filtration step. First, Rt reproducibility and mass accuracy were evaluated. Minimal shifts were observed between the theoretical and experimental retention times of both the internal standards and the unlabeled analytes. Additionally, there were minimal mass errors detected (an average of <5 ppm), indicating high mass accuracy (Figure S1). To evaluate the quality and accuracy of the data, a normalization step was conducted using the systematical error removal using random forest (SERRF) method. This resulted in a reduction of the variation represented by RSD % from 39.18 to 26.18% (Figure S2). In parallel, the data was treated without normalization, but with feature filtering based on the Rt and %CV of the QC samples, leading to a total of 1238 features. Comparison of the PCA scores plots derived with and without the SERF normalization, but in the latter case with data filtering, did not demonstrate significant differences (Figure S3). Hence, the un-normalized data was used for further analysis.

**Figure 1:**
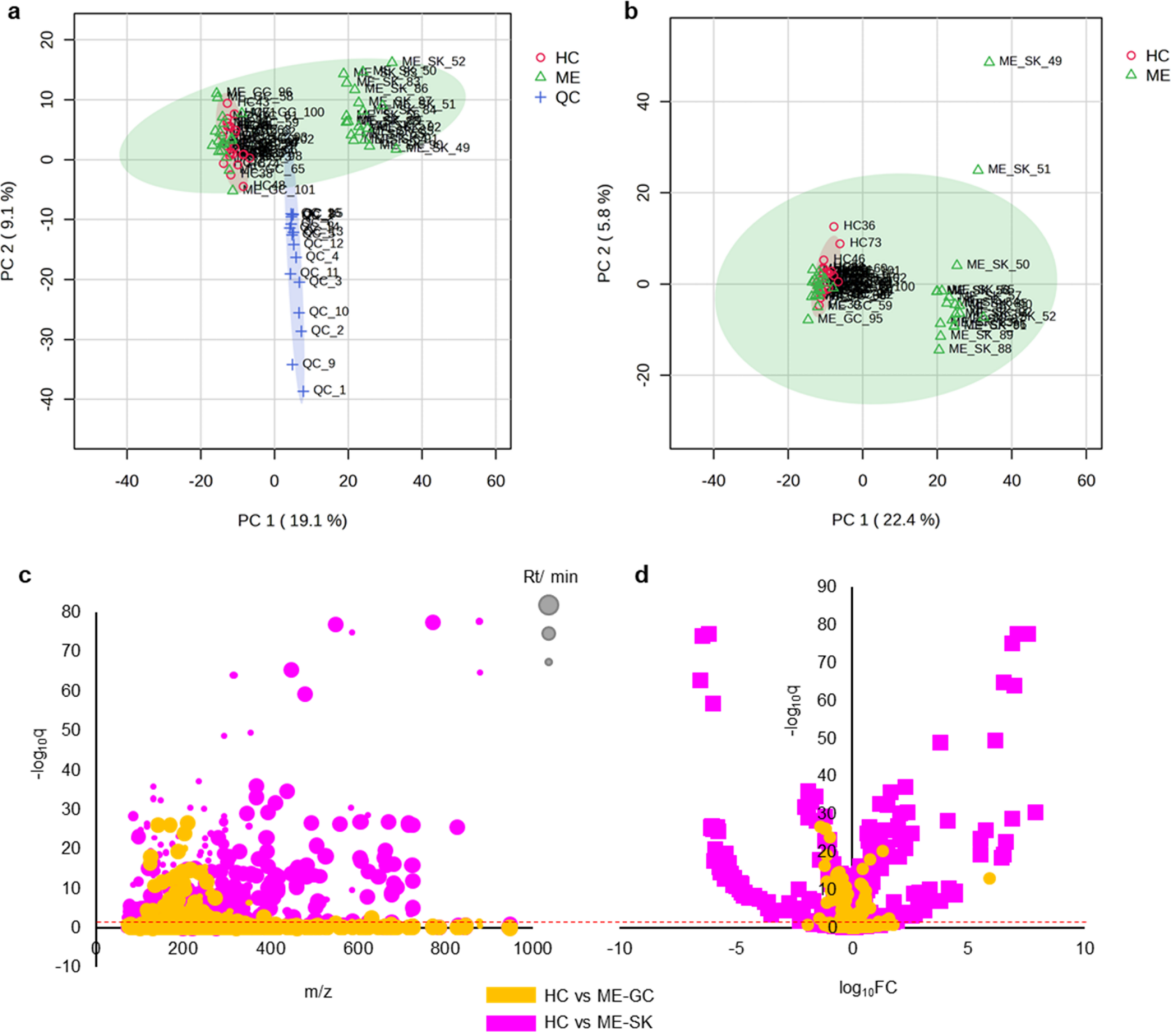
PCA plots are presented to show the distribution of QC, HC, and ME samples. Panel (a) illustrates PC1 (19.1% variance) and PC2 (9.1% variance), with QC samples clustering centrally along PC1 but separating along PC2. Panel (b) highlights that the Stockholm (SK) cohort of the ME/CFS patients is distinct from other plasma samples. Panels (c, d) depict significant metabolite changes, with 115 features differentiating HC and ME/CFS patients from Gothenburg cohort (ME-GC), and 522 features differentiating HC and ME-SK, corroborating the PCA’s indication of high discrimination in the ME-SK cohort.

The scores plot of the first (PC1) and second (PC2) components displayed a reasonable distribution of the QC samples, which were located in the center of PC1, the component explaining the highest variance in the data (19.1%). However, the QC samples were separated from the rest of the samples on PC2, explaining 9.1% of the total variance (Figure 1a). The highest variance in the data, nonetheless, was found between the SK cohort and the rest of the plasma samples (Figure 1a,b), which might be due to the heterogeneity of the disease and the impact of different comorbidities. Due to the significant difference between the two ME cohorts, clearly reflected on the unsupervised scores plots, the screening for metabolic changes induced by ME was performed in the two cohorts independently. From this perspective, common significantly altered metabolites reflect disease-related modifications impartially from sample collection bias. When comparing the HC samples to the ME-GC cohort, 115 features were found to be significantly different, while when comparing to the ME-SK cohort, 522 features were significantly different (Figure 1c,d). This difference was expected based on the PCA scores plot, which indicated a high degree of discrimination regarding the ME-SK cohort. The significantly altered features from both comparisons were cross-validated and there were 30 common features identified (Table S1) and (Figure S4). Metabolic annotation of these features was initially performed using the HMDB database, based on the high mass accuracy. Among the significant features, the tryptophan metabolic products quinolinic acid and indoleacetic acid were detected with high identification confidence level,^11,12^ both being downregulated in ME patient samples (Table S1 and Figure S4).

Untargeted metabolic pathway analysis was performed using the total 1238 features for HC vs ME-GC and HC vs ME-SK, separately (Figures S5 and S6). A number of metabolic pathways were found to be commonly disregulated between the two analyses, especially the vitamin B3 and the arginine-proline and aspartate-asparagine pathways. Also, l-Adrenaline and S-adenosyl-l-homocysteine (SAH) are linked in the metabolic pathway analysis for both HC vs ME-GC and HC vs ME-SK. Adrenaline is known to inhibit SAH hydrolase, which stops the conversion of SAH to homocysteine, therefore halting the methylation cycle.^13^ Alterations in the methylation cycle of ME/CFS patients have been proposed before and there has been focus on mutations in *MTHFR* in this patient population.^14,15^ Furthermore, adrenaline has been shown to be an important factor in ME/CFS and postural orthostatic tachycardia syndrome (POTS) is a common comorbidity of ME/CFS where adrenaline plays an integral role.^16^ More recently, the aspartate-asparagine pathway and the arginine-proline pathway were highlighted as being anomalous in ME/CFS and Long COVID populations when compared to healthy controls.^17^ Again here, both ME-SK and ME-GC populations displayed anomalies in both the aspartate-asparagine pathway and the arginine-proline pathway, highlighting a potential important and consistent area of metabolic focus in ME/CFS.

### 2.2 Quantitative Analysis of Tryptophan Metabolites and Related Compounds

To verify if similar variance is observed in the targeted analysis, we also checked the PCA for HC and ME cohorts (Figure S7.A) and PCA for ME-GC and ME-SK cohorts (Figure S7.B) to see if there are differences, as we noticed in the untargeted method. The PCA plots (Figure S7.A,B) reveal some distinct differences within the ME cohort, suggesting that the metabolic impact of ME varies between the subcohorts ME-GC and ME-SK. A Wilcoxon test was conducted to identify the analytes responsible for these findings, revealing significant differences (*p* < 0.05) in hypoxanthine, phenylalanine, 5-HT, riboflavin, and nicotinamide between HC and ME-SK; 5-HT, hydroxyanthranilic acid, hypoxanthine, and nicotinamide between HC and ME-GC; and phenylalanine, hypoxanthine, 5-HT, and riboflavin between ME-GC and ME-SK. The linear model with covariate adjustments was selected to assess the significance of sex on analyte concentration, with cohort type (HC vs ME) and age included as covariates to control for their effects. While, the Pearson correlation coefficient (Pearson r) was used to assess the correlation between analytes and age, controlling for cohort type (HC vs ME) and sex (Table S2). These results indicate significant correlations for kynurenine with both sex (*p* = 0.001) and age (*r* = 0.48, *p* = 0.0001), and for quinolinic acid with both sex (*p* = 0.046) and age (*r* = 0.42, *p* = 0.001), suggesting potential biological differences or age-related changes in these metabolites. Additionally, kynurenic acid is significant with sex (*p* = 0.003), hydroxyanthranilic acid with age (*r* = 0.29, *p* = 0.02), and hydroxykynurenine with age (*r* = 0.43, *p* = 0.001).

**Figure 2:**
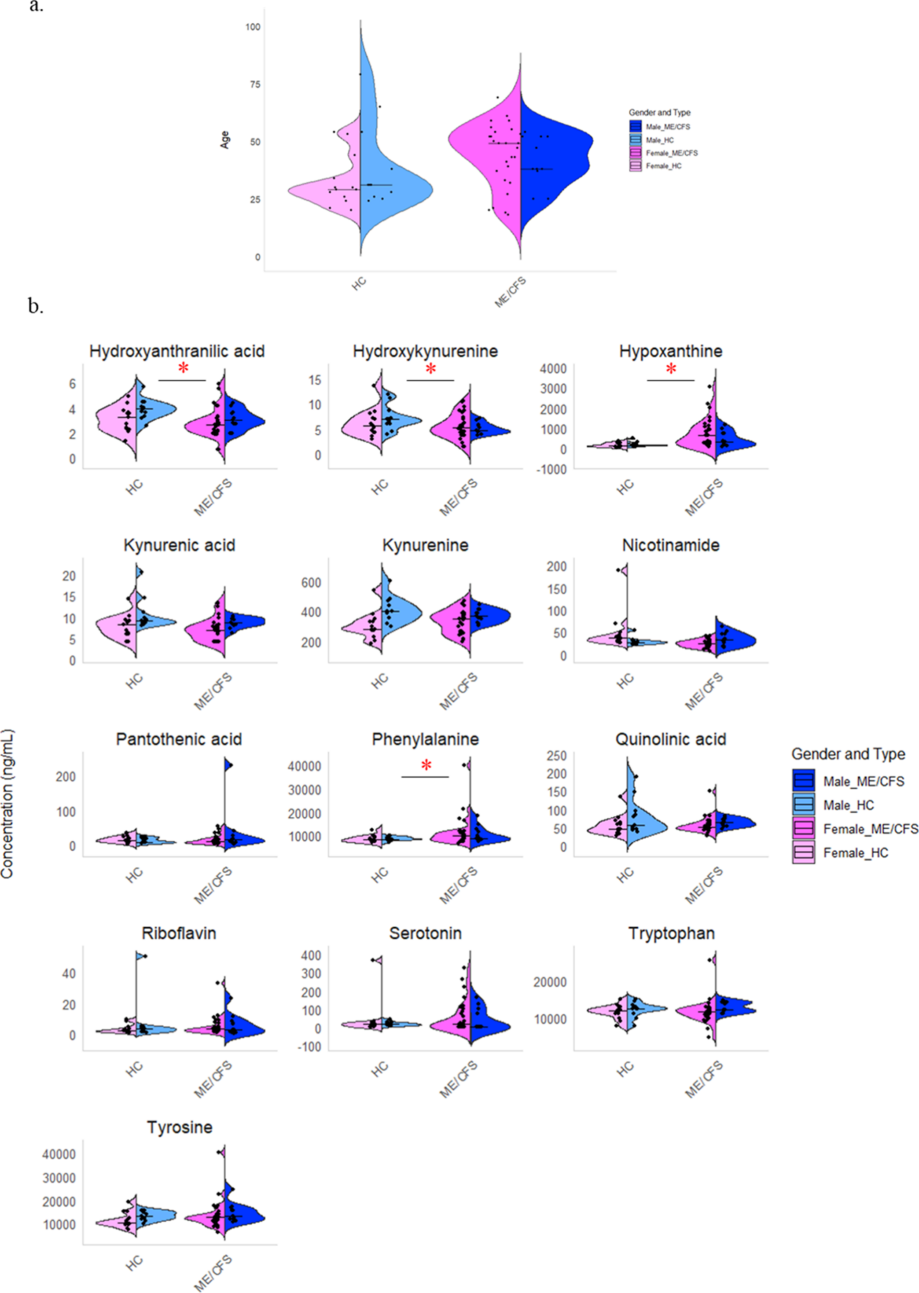

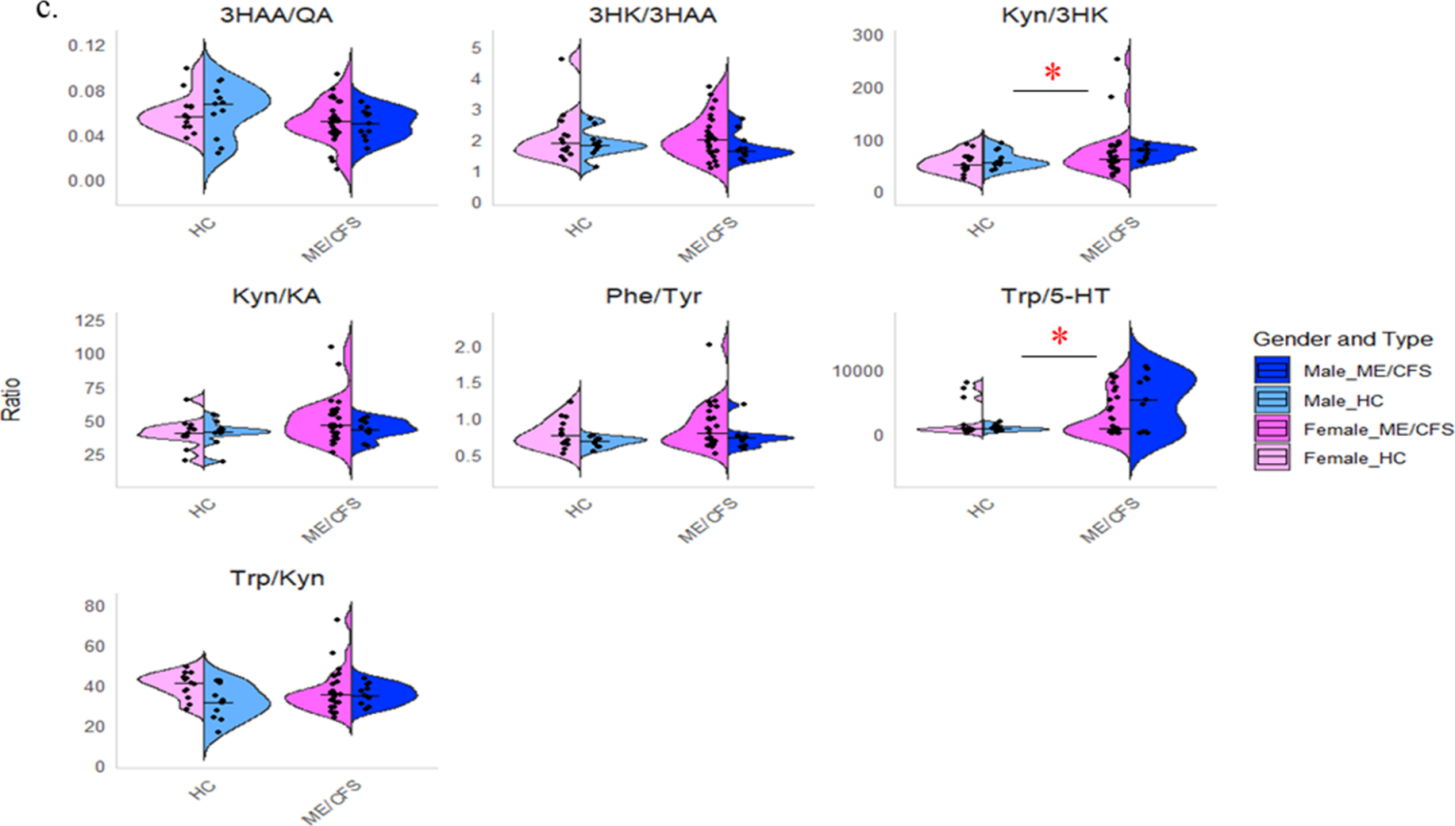
Violin plots of ME/CFS and healthy controls (HC) separated into males and females. (a) A violin plot of age distribution for 24 presumed HC, (13 females and 11 males) and 38 ME/CFS patients (27 females and 11 males), separated by sex. The *x*-axis represents the cohorts, and the *y*-axis represents age. Each violin indicates the density of participants at different ages, with individual ages shown as dots and median age as horizontal lines. Both cohorts span a wide age range, with more participants in the middle-age range, and show sex-specific distribution patterns. (b) Presents violin plots showing the concentration distributions of various metabolites for HC and ME/CFS patients, divided by sex. Each subplot represents a different metabolite. Notably, phenylalanine and hypoxanthine concentrations are significantly higher in ME/CFS patients, while hydroxyanthranilic acid and hydroxykynurenine concentrations are lower in ME/CFS patients compared to HC. (c) Presents violin plots displaying the ratios of 3-hydroxyanthranilic acid to quinolinic acid (3HAA/QA), 3-hydroxykynurenine to 3-hydroxyanthranilic acid (3HK/3HAA), kynurenine to 3-hydroxykynurenine (Kyn/3HK), kynurenine to kynurenic acid (Kyn/KA), phenylalanine to tyrosine (Phe/Tyr), tryptophan to kynurenine (Trp/Kyn) and tryptophan to serotonin (Trp/5-HT). Notably, the ratios of Kyn/3HK and Trp/5-HT are higher in the ME/CFS cohort compared to HC. The *x*-axis shows the cohorts, and the *y*-axis shows metabolite concentration (ng/mL) or ratio. Individual data points are overlaid as dots, and horizontal lines indicate median concentrations. The red star * represents (*p* < 0.05).

The levels of tryptophan metabolites, along with phenylalanine, tyrosine, riboflavin, pantothenic acid, and hypoxanthine, in plasma samples collected from 38 ME/CFS patients and 24 healthy controls (HC), are illustrated in the ME/CFS-HC analysis (Figure 2b). The median and interquartile range (IQR) for each analyte, stratified by health status and sex, are presented in Table 1, while the ratios for some of the metabolites are detailed in (Table S3). We evaluated these concentrations based on sex due to notable variances in serotoninergic changes and tryptophan metabolism between males and females.^18^ We observed that the concentration of **3-hydroxykynurenine (3HK)** and **3-hydroxyanthranilic acid (3HAA)**, a metabolite of 3HK, were significantly lower in ME/CFS patients compared to the control group with a p-value of 0.003 and 0.021 respectively. 3HAA is vital for immune responses and its regulation could have significant health implications. It controls cytokine release from T helper cells and hinders the transcription factor NFκB and nitric oxide synthase, affecting T cell function.^19^ Our study aligns with Kavyani et al.’s findings on reduced 3-hydroxykynurenine levels. However, we diverged from their results regarding 3-hydroxyanthranilic acid, where they reported no significant differences between ME/CFS patients and control plasma.^10^ By examining the tryptophan metabolite ratios shown in Figure 2c, we found that the kynurenine/3-hydroxykynurenine (Kyn/3HK) ratio is elevated in ME/CFS, aligning with our finding that 3HK is decreased in the ME/CFS cohort.

**Table 1:** Plasma Levels of Tryptophan Metabolites, Hypoxanthine, Pantothenic Acid, Phenylalanine, Riboflavin and Tyrosine, Reported as Median and Inter Quartile (IQR) in mg/mL, for Healthy Control (HC) and Myalgic Encephalomyelitis/Chronic Fatigue Syndrome (ME/CFS) Patients

analyte		HC		ME/CFS		HC vs ME/CFS
	gender	median	IQR	median	IQR	*p*-value
**hydroxyanthranilic acid**	female	3.3	2.3–3.6	2.7	2.1–3.2	**0.021**
	male	3.9	3.5–4.3	3.0	2.7–3.6	
**hydroxykynurenine**	female	5.7	4.3–7.1	5.3	4–7.2	**0.003**
	male	7.0	6.1–7.9	4.6	4.5–5.8	
**hypoxanthine**	female	128.3	73.1–236.6	640.9	218.6–1075.2	**0.002**
	male	134.3	106.6–147.5	285.0	151.6–725.5	
kynurenic acid	female	8.1	6.1–9.1	6.9	4.2–8.1	0.064
	male	9.2	8.8–10.5	8.7	8.2–9.6	
kynurenine	female	280.3	272.4–327.6	345.4	262.4–384.9	0.218
	male	398.6	353.8–452.1	366.4	336.1–399.5	
nicotinamide	female	36.1	29.8–43.9	24.0	17.6–29.6	0.052
	male	27.2	24.7–30.1	33.7	22.2–44.6	
**pantothenic acid**	female	14.4	11.1–23	9.6	6.4–19.3	**0.310**
	male	8.8	7.9–20.4	17.5	9.1–27.5	
phenylalanine	female	8145.9	7575.3–9553.5	10067.1	8067.2–11577.3	0.035
	male	8391.8	8318.3–9291.7	8728.4	8278.1–12030	
quinolinic acid	female	45.0	41.8–63.5	52.1	44.8–63.9	0.087
	male	55.7	46.6–90.3	62.8	54.8–73.2	
riboflavin	female	2.7	1.8–3.9	3.5	2.3–6.2	0.739
	male	3.6	1.9–4.6	3.1	1.5–8.1	
serotonin	female	16.5	7–27.3	16.9	2.2–91.6	0.535
	male	17.5	11.7–24.7	2.4	1.4–87.7	
tryptophan	female	12017.4	11008.3–12550.6	11564.5	10304.1–12642.2	0.394
	male	12382.7	11653.5–13148.2	12253.8	11881.1–14146.9	
tyrosine	female	10408.4	9606.1–12201.6	12544.3	10778.7–13948.6	0.250
	male	13065.0	12082.3–15025.9	13070.0	11812.7–16171.4	

It is noteworthy to emphasize that prior studies on ME/CFS metabolomics mainly focused on untargeted metabolomics,^6^ a small number of TRP metabolites,^5,20,21^ or investigated organic and amino acids without incorporating TRP.^22,23^ Our study enhances these previous findings by providing a more comprehensive and quantitative measurement of the TRP pathway, resulting in a more extensive and precise analysis using high-resolution mass spectrometry method. Furthermore, we accounted for the influence of participants’ age and sex, which has been a prevalent limitation in previous studies. These developments not only improve the accuracy of our findings but also enable a more profound comprehension of the physiological and clinical importance of the observed variations in comparison to previous research.

Additionally, the tryptophan/serotonin (Trp/5-HT) ratio (Table S3) is elevated in male ME/CFS patients indicating a reduction in the enzymes that convert tryptophan to 5-HT, which might be important to disease progression in males with ME/CFS. Tryptophan hydroxylase is the rate limiting step of converting tryptophan to 5-HT and it requires BH_4_, an important cofactor hypothesized to be elevated in ME/CFS.^24^ Notably, this elevated Trp/5-HT ratio in male patients is approximately seven times higher than in female patients. This significant difference emphasizes the importance of considering sex-related factors, as serotonin levels can be influenced by sex.^18,25,26^ However, our study is limited by the lack of information on the menstrual cycle phase of the female participants and any medications they were taking.

**Hypoxanthine** is a naturally occurring purine derivative involved in nucleic acid metabolism. In hypoxic conditions, where oxygen supply to tissues is reduced, hypoxanthine levels can rise due to increased ATP breakdown.^27^ Consequently, hypoxanthine serves as a biomarker for cellular hypoxia, which may be relevant to reduced oxygen extraction in ME/CFS.^28^ In our study, elevated hypoxanthine levels in ME/CFS patients compared to HC, (*p* = 0.002) suggests a potential link between hypoxia and ME/CFS pathology. This finding aligns with the understanding that ME/CFS patients often exhibit metabolic dysregulation, leading to cellular stress and hypoxic conditions, which correlate with symptoms like fatigue and reduced energy metabolism.^7,29^ In a study by Shida et al. they found that hypoxanthine disrupts muscle energy metabolism by reducing ATP levels crucial for muscle contraction.^30^ This disruption activates uncoupling protein 2 (UCP2), leading to mitochondrial decoupling and muscle weakness so elevated hypoxanthine levels in ME/CFS patients, may exacerbate muscle degradation. It is worth noting that the recurring presence of hypoxanthine as a metabolite varies significantly between studies. Furthermore, Naviaux et al. have proposed that the elevated efflux of purine metabolites may be a stress signal that propagates reduced energy production in ME/CFS.^31^

Another altered analyte in our cohort is **phenylalanine**. Its levels were found to be significantly elevated in ME/CFS patients compared to HC, with a *p*-value of 0.035. This aligns with previous research indicating altered amino acid utilization in ME/CFS. Initial findings by Xu et al. indicated elevated phenylalanine levels in peripheral blood mononuclear cells of ME/CFS patients using a single-cell Raman platform.^32^ However, a subsequent study with a larger cohort revealed decreased levels.^33^ Additionally, Armstrong et al. observed decreased phenylalanine levels compared to healthy controls,^6^ which contradicts with our findings. Lastly, Figure S8 summarizes the four altered analytes and illustrates their distribution across different age groups within the cohorts.

### 2.3 Comparison of Semiquantitative versus Quantitative Analysis

To compare our findings from 38 ME/CFS patients and 24 HC, we utilized an eight-point calibration curve. Additionally, we estimated the sample concentrations by determining the area ratio of each analyte to the corresponding internal standard, as outlined in our previous publication, and then multiplied by the internal standard concentration (semiquantitative).^34^ Subsequently, we correlated the concentrations obtained through quantitative and semiquantitative methods and we calculated the difference between them. The results are depicted in (Figure S9). Notably, most analytes exhibited a correlation coefficient exceeding 0.995, with hypoxanthine being the exception (*R*^2^ = 0.806). Upon closer examination of the method comparison, we observed discrepancies lower than 20%. It is noteworthy that when we use internal standards other than the isotope-labeled versions of the target compounds, we observe higher deviations between the two methods. For instance, hypoxanthine, utilizing quinolinic acid-[^13^C_4_,^15^N], as well as pantothenic acid and riboflavin, utilizing theobromine and biotin-[^2^H_2_] respectively, displayed notable discrepancies. Furthermore, the difference between the two methods was pronounced for 5-HT at lower concentrations, close to the lower limit of quantification (LLOQ). However, this difference became less pronounced as concentrations increased. This highlights the utility of semiquantitative analysis in metabolomics, providing results comparable to quantitative methods employing external calibration curves. This approach proves cost-effective and time-efficient, enabling the evaluation of metabolite relative abundance in samples.

## 3 Conclusions

The untargeted analysis reveals significant alterations in several metabolic pathways, including the vitamin B3, arginine-proline, and aspartate-asparagine pathways, while the targeted analysis highlights changes in two key compounds within the tryptophan pathway, namely 3HK and 3HAA, which may be linked to altered immune responses. Additionally, the elevated Trp/5-HT ratio in ME/CFS patients indicates lower serotonin levels, potentially contributing to the mental and sleep disorders observed in this cohort. We also found changes in the oxidative stress marker hypoxanthine and the amino acid phenylalanine. Significant differences between the ME subcohorts, ME-GC and ME-SK, were observed in both untargeted and targeted analyses. Additionally, significant correlations for both kynurenine and quinolinic acid with sex and age suggest potential biological differences or age-related changes in these metabolites. The limitations of this study include the absence of data on body mass index (BMI), participants’ diets, medications, and the menstrual cycle phases of female participants, all of which could potentially influence TRP and its metabolite levels. This study also highlights the value of semiquantitative analysis, demonstrating that it provides results comparable to quantitative methods using external calibration.

## 4 Methods

The analytical method employed is the same as in our previous study with more detailed information regarding the chemicals and internal standards utilized.^34^

### 4.1 Sample Collection and Preparation

Human plasma samples were collected from 38 patients with ME/CFS, consisting of 27 females and 11 males, with an average age of 43 years (±13). These patients were recruited between 2013 and 2018 from two clinics: Stora Sköndal in Stockholm (SK), where the cohort included 19 patients (15 females and 4 males) with an average age of 44 years (±15), and Gottfries Clinic in Gothenburg (GC), which included 19 patients (12 females and 7 males) with an average age of 43 years (±12).

The SK and GC samples were collected after obtaining approval from the regional ethics committees in Stockholm (2016/4:7) and Göteborg (2016:966–15) respectively. Both groups were diagnosed using Canadian Consensus Criteria, the International Consensus Criteria, and the Institute of Medicine (IOM) criteria. Infection was the trigger of ME/CFS in 87.5% of the Stockholm group while, no information was available regarding the trigger for the Göteborg group. Those cohorts have been further examined for the presence of autoantibodies, in the published study by Bynke et al.^35^ According to reference (33), there were 48 patients in total. However, in this current investigation, we only employed 38 individuals in total, as 10 of the samples were of insufficient volume for our analysis. Additionally, plasma samples were collected from 24 presumed healthy donors (13 females and 11 males) with an average age of 36 (±15) yrs. These donors are employees in the Department of Analytical Chemistry at Uppsala University. All samples were collected between 9 and 11 a.m., following a 12-h fast, except for three participants who were not fasting. Verbal and written consent were obtained from each donor, and the study was approved by the Ethical Review Agency (Dnr 2021–02859). A pooled sample was created for quality control (QC) purposes for the untargeted analysis by combining 20 μL of each plasma sample from patient and healthy control (HC). Figure 2a summarizes the demographic information for the HC and ME/CFS cohorts showing the sex and age distribution.

An aliquot of 75 μL of each sample, pooled QC and blank along with 25 μL of the internal standard working solution was transferred to an Eppendorf tube. To initiate protein precipitation, 25 μL of 0.1% formic acid was added to each sample, which was then vortexed before, 375 μL of cold methanol was subsequently added. The solution was vortexed for 15 s at 1600 rpm and stored at −20 °C for 30 min. The samples were then centrifuged at 10,000*g* for 10 min at 4 °C, and the supernatant was collected and concentrated using a nitrogen stream supplied by TurboVap LV (Biotage, Uppsala, Sweden) at a flow rate of 1.2 mL/min at 25 °C then it was reconstituted in 100 μL of 0.1% formic acid. After being vortexed for 15 s and HT centrifuged at 10,000*g* for 10 min, the supernatant was analyzed by LC-HRMS.

### 4.2 LC-HRMS Analysis

A Waters Acquity UHPLC was coupled to a high-resolution Q Exactive hybrid quadrupole-Orbitrap mass spectrometer to perform analyses. The LC column was a Waters HSS T3 (1.8 μm: 2.1 × 100 mm^2^) kept at 30 °C. Data acquisition and processing were done by Xcalibur software (Version 4.1, Thermo Scientific), while Tune interface software (Tune v2.9) was used for tuning and optimizing the analytes. The mobile phases consisted of Milli-Q water plus 0.6% formic acid (100:0.6%, v/v) (Mobile Phase A), and methanol plus 0.6% formic acid (100:0.6%, v/v) (Mobile Phase B). The gradient was 1–10% B (0.0–4.0 min), 10–90% B (4.0–7.0 min), hold at 90% B (7.0–8.0 min), 90–1% B (8.0–8.1 min) followed finally, by equilibration at 1% B (8.1–9.0 min). For the untargeted analysis, a full scan (*m*/*z* 67–1000) was performed at a resolving power of 70,000 full width at half-maximum (FWHM) at *m*/*z* 200. Data-dependent MS/MS were acquired in “Top5” mode with a resolution of 17,500 FWHM in positive electrospray ionization (ESI+) mode. The samples for untargeted analysis were injected to the LC-HRMS system in a randomized order with QC samples injected in the beginning, after every tenth sample and end of the sample list. For the targeted analysis, we used a timed parallel reaction monitoring (PRM) method with the same LC-HRMS parameters as in our previous study^34^

### 4.3 Data Analysis

#### 4.3.1 Data Extraction and Processing

The **untargeted** analysis was performed using XCMS online, for the chromatograms and mass spectra processing through alignment and time correction. To evaluate the stability and performance of the experimental setup and instrument over time, the QC intensities were plotted against the LC-HRMS sample injection order. The mass accuracy between the theoretical and experimental values (ppm) and the retention time (Rt) reproducibility were evaluated using labeled standards. The impact of data normalization was examined by analyzing the data with and without SERRF normalization.^36^ In the latter case, features with 1 min ≤ Rt ≤ 7.5 min, and % CV of the QC’s < 40% were selected for statistical analysis after log_10_ transformation of the mass intensity values. In both approaches, the Metaboanalyst software 6.0 was utilized to give an overview of the data by principle component analysis (PCA) after autoscaling.^37^ PCA provided an unsupervised insight of the data, i.e., it identified potential outliers and exploration of the main variance, which was utilized as a guidance for subsequent hypothesis testing. From this perspective, multiple *t* tests between the healthy control and each of the myalgic encephalomyelitis cohorts (ME-GC and ME-SK, respectively) were performed with *q*-value correction (*q* < 0.05). Subsequently, the common significant features were selected and the direction of the effect (up- or down-regulation) was examined to be the same. This approach served as a latent “test set” validation, further restraining the probability of false positives and compensating for the limited sample size. In addition, the correlation between the cross-validated significant features and confounding factors such as age and sex were calculated (Pearson’s coefficient, *r*). Pathway analysis was performed using the Mummichog software.^38^ The analysis focused on the possible metabolic pathways involved in ME/CFS by independently comparing the HC group with each of the ME cohorts. For metabolite identification, significant features were primarily annotated by databases (www.hmdb.ca, GNPS) based on their *m*/*z* value and given the high mass accuracy provided by the mass analyzer.^39,40^ Form HMDB search, a threshold of 5 ppm was applied and the adducts [M + H], [M + H – H_2_O], [M + Na] and [M + K] were selected. In addition, Rt values from analyzed standards were used for structural validation together with MS/MS spectra acquired by the “Top5” mode.

The data analysis for the **targeted** section has been performed in the same manner as in our previously published article using Xcalibur software (Version 4.1, Thermo Scientific).^34^ For specific metabolites (3-hydroxyanthranilic acid, 3-hydroxykynurenine and 5-HT), few values were below the lower limit of quantification (LLOQ) and were imputed as LLOQ/√2 for analysis.^41^ Statistical analyses were conducted using RStudio 2024.04.1 (r-project.org). We applied a log transformation to the analyte concentrations as the metabolites deviated from a normal distribution, as assessed by the Shapiro-Wilk normality test. Subsequently, we performed a linear mixed-effects model to analyze the impact of health status (HC versus ME/CFS), sex, and age on metabolite concentrations, with analyte included as a random effect. Then, we conducted an ANOVA type III to test the significance of these predictors and identified which analytes showed significant differences between HC and ME/CFS after correcting for age and sex.

## Supporting Information Available

Detailing structure elucidation; statistical analysis; significant features for the untargeted analysis; untargeted metabolic pathway analysis (PDF)
