# Supplementary material for: Untargeted Metabolomics and Quantitative Analysis of Tryptophan Metabolites in Myalgic Encephalomyelitis Patients and Healthy Volunteers: A Comparative Study Using High-Resolution Mass Spectrometry
Source: ACS Chem Neurosci. 2024 Sep 20;15(19):3525–34. doi: 10.1021/acschemneuro.4c00444 (PMC11450765; doi:10.1021/acschemneuro.4c00444)
Supplement: Supplementary file 1 — cn4c00444_si_001.pdf [file cn4c00444_si_001.pdf]

Supporting Information

Untargeted metabolomics and quantitative analysis of tryptophan metabolites in myalgic encephalomyelitis patients and healthy volunteers: a comparative study using high resolution mass spectrometry

Sandy Abujrais[1,2], Theodosia Vallianatou[3], Jonas Bergquist[1,2]

[1]*Analytical Chemistry and Neurochemistry, Department of Chemistry – BMC, Uppsala University, Box 599, 751 24 Uppsala, Sweden*

[2]*The ME/CFS/CFS Collaborative Research Centre at Uppsala University, Sweden*

[3]*Spatial Mass Spectrometry, Department of Pharmaceutical Biosciences, Uppsala University, Box 591, 751 24 Uppsala Uppsala, Sweden*

Corresponding author email address: jonas.bergquist@kemi.uu.se

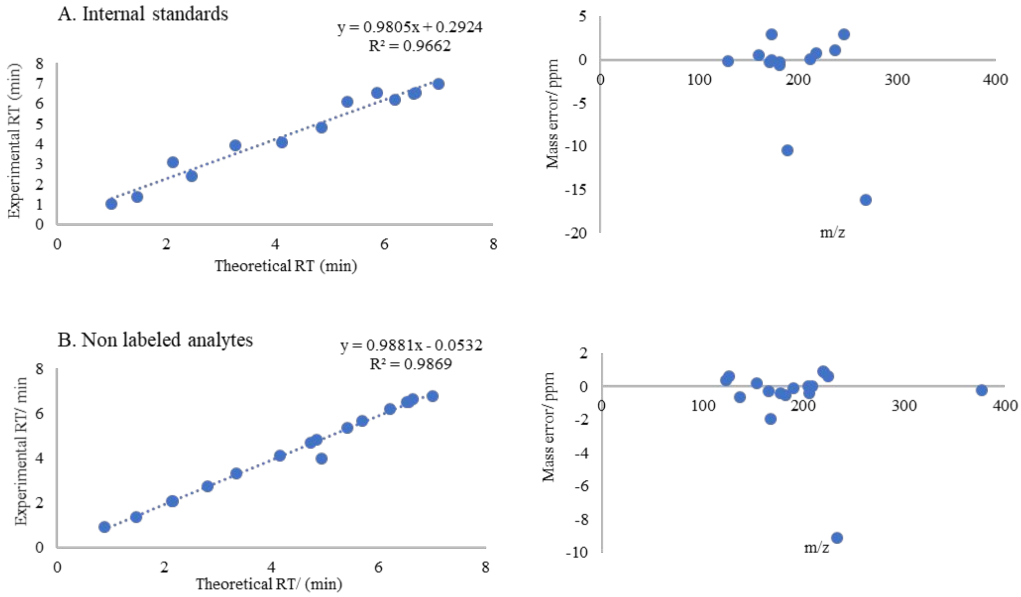

Figure S1. A) Illustrates the correlation between the theoretical and experimental retention times of the internal standards. The theoretical retention time is determined by injecting the internal standards into the LC-MS instrument independently from the samples, while the experimental retention time is obtained by adding the internal standards to ME/CFS and HC plasma, running untargeted analysis, and extracting the retention times from XCMS output using the Rt median. Additionally, we plotted the mass-to-charge ratio against the mass error for these internal standards. B) Presents a similar plot, but for the unlabeled analytes.

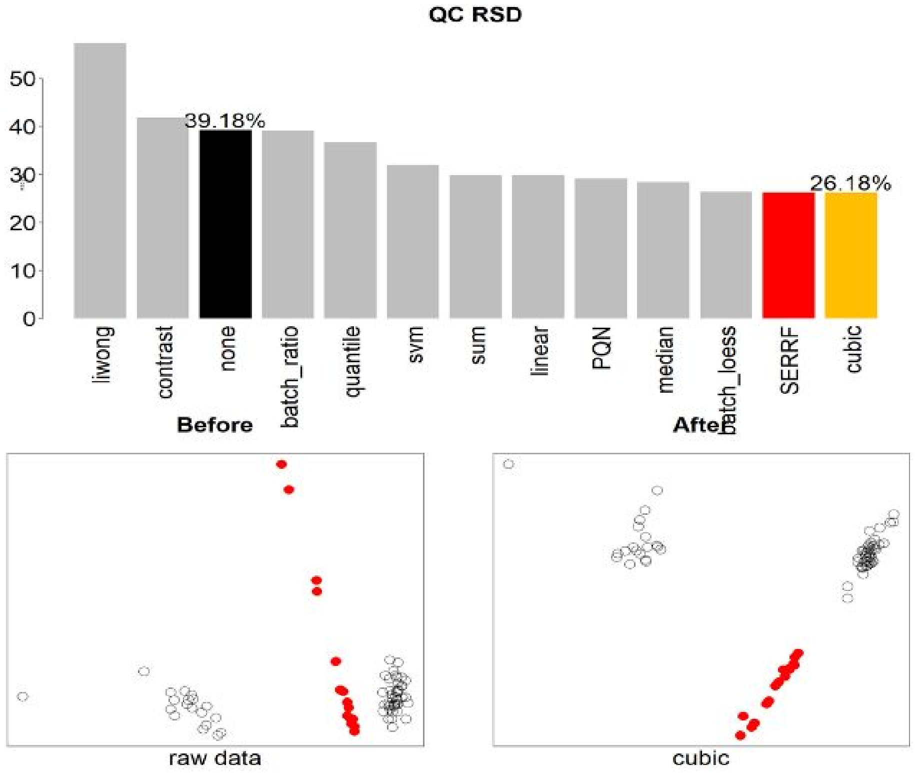

Figure S2. The impact of normalization based on pooled QC's by SERRF method on RSD % values. Before normalization, RSD % was 39.18%, indicating high variation. After normalization, RSD % dropped to 26.18%, showing reduced variation and improved data quality. This visual representation demonstrates the effectiveness of normalization in standardizing data and enhancing measurement accuracy.

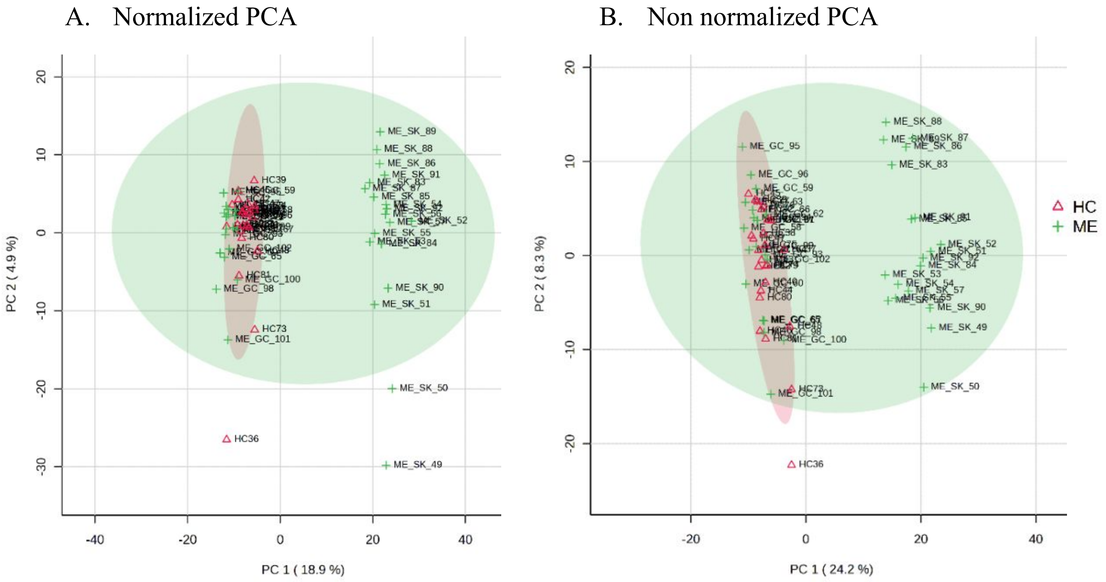

Figure S3. A) The normalized PCA plot demonstrates the distribution of data points after undergoing a normalization process using the SERRF method, which removes unwanted variations. It provides an unbiased visual representation of patterns and main sources of variation in the dataset. B) The non-normalized PCA plot compares the metabolomic profiles of healthy control (HC) individuals and those with myalgic encephalomyelitis (ME), showing inherent differences between the two cohorts.

Table S1. A list of the significant features with their mass and retention time (RT). It includes their correlation with age and examines the impact of sex. Additionally, it provides the significance levels for comparisons between Healthy Controls (HC) versus ME patients from Gothenburg cohort (GC) and between HC versus ME patients from Stockholm cohort (SK).

ID	*m/z*	Rt (min)	Age correlation (Pearson's r)	Sex significance (raw P value)	HC vs GC (q value)	HC vs GC (logFC)	HC vs SK (q value)	HC vs SK (logFC)	Annotation	Confidence level [11, 12][a]
M227T1_6	227.1252	1.01	-0.002	0.008	1.44E-02	0.15	3.06E-02	0.15	$C_8H_{15}O_2N_6$	4
M249T1_5	249.0283	1.06	-0.030	0.003	4.73E-04	0.23	4.87E-02	0.11	$C_{12}H_{30}O_2N_5$	4
M233T1_6	233.0508	1.07	0.011	0.063	4.12E-02	0.24	1.03E-03	1.63	$C_5H_{13}O_{10}$	4
M239T1_3	239.0639	1.07	-0.253	0.228	9.94E-09	0.52	4.36E-19	6.41	$C_7H_{13}O_8N$	4
M217T1_5	217.0820	1.10	-0.192	0.090	1.22E-07	0.48	4.60E-31	2.37	$C_8H_{13}O_5N_2$	4
M173T1_4	173.0920	1.13	-0.156	0.427	9.94E-09	0.60	3.14E-13	0.74	$C_7H_{13}O_3N_2$	4
M249T1_7	249.1082	1.13	-0.252	0.057	3.75E-16	0.44	1.27E-21	2.28	$C_9H_{17}O_6N_2$	4
M205T1_7	205.1182	1.15	-0.239	0.233	7.03E-21	1.32	2.78E-16	1.24	$C_8H_{17}O_4N_2$	4
M263T1_6	263.1239	1.29	-0.248	0.034	2.76E-10	0.60	1.17E-24	1.13	$C_{10}H_{19}O_6N_2$	4
M351T1_4	351.1032	1.31	-0.145	0.053	4.07E-07	0.41	1.83E-26	5.75	$C_{11}H_{13}O_5N_9$	4
M307T1_7	307.1137	1.36	-0.179	0.145	5.37E-05	0.48	6.83E-20	6.51	$C_{11}H_{19}O_8N_2$	4
M217T2_2	217.0820	1.57	-0.209	0.172	1.61E-04	0.39	2.43E-07	1.81	$C_8H_{13}O_5N_2$	4
M168T2	168.0288	2.09	0.051	0.248	8.69E-03	0.33	8.99E-03	0.28	Quinolinic acid	1
M263T2_3	263.1602	2.16	0.222	0.041	1.50E-03	-0.30	6.67E-04	-0.28	$C_{10}H_{17}N_9$	4
M234T2_2	233.9823	2.22	-0.159	0.531	3.81E-02	0.20	3.57E-07	0.54	$C_{13}O_4N$	4
M130T2_2	130.0499	2.23	0.007	0.456	6.26E-03	-0.08	1.32E-06	-0.35	Pyroglutamic acid (5-oxo-proline)[b]	2a
M230T2_1	229.9748	2.25	0.108	0.576	1.50E-04	-0.21	4.37E-10	-0.54	$CH_3O_{10}N_4$	4
M275T2	274.9723	2.25	0.131	0.201	2.86E-02	-0.19	2.29E-09	-0.59	$HO_{11}N_7$	4
M219T5_1	219.0436	5.44	-0.083	0.174	5.42E-06	0.77	1.83E-03	1.40	$C_{15}H_7O_2$	4
M120T7_2	120.0558	6.63	-0.140	0.284	1.93E-04	0.20	1.94E-02	0.10	$C_6H_6N_3$	4
M198T7_2	198.1276	6.71	0.203	0.985	1.93E-04	-0.27	4.20E-05	-0.31	$C_{10}H_{16}O_3N$	4
M170T7_3	170.1175	6.80	0.187	0.850	9.40E-27	-1.20	2.92E-02	-0.09	$C_9H_{16}O_2N$	4
M124T7_2	124.1122	6.80	0.204	0.614	5.32E-17	-1.18	9.10E-09	-0.52	$CH_{12}ON_6$	4
M226T7_3	226.1416	6.82	0.185	0.910	2.18E-07	-1.12	4.49E-04	-0.50	$C_{10}H_{16}O_3N_3$	4
M172T7_2	172.1332	6.82	0.194	0.905	5.67E-13	-1.12	1.01E-04	-0.20	$C_9H_{18}O_2N$	4
M176T7_3	176.0706	7.11	0.060	0.110	2.79E-02	0.14	2.35E-07	0.33		
M130T7_2	130.0652	7.11	0.066	0.092	1.65E-02	0.14	1.21E-07	0.32	Indoleacetic acid[c] $C_{12}H_8O_5N_2$	2a
M260T7_1	260.0233	7.11	0.064	0.197	3.64E-03	0.21	1.41E-05	0.40		
M198T7_1	198.0524	7.11	-0.066	0.090	1.40E-02	0.15	1.89E-14	0.69	Indoleacetic acid [M+Na]	2a
M276T7_1	275.9957	7.11	0.107	0.237	7.54E-03	0.17	4.10E-04	0.22	$C_{16}H_6O_4N$	4

a. Level 5, exact mass; level 4, molecular formula; level 3, *in silico* MS/MS; level 2b, diagnostic fragments or qualifier ions; level 2a, MS/MS spectra from libraries; level 1, MS/MS and Rt from reference standard
b. GNPS Library Spectrum CCMSLIB00005883999- common product ion *m/z* 84.0445
c. GNPS Library Spectrum CCMSLIB00005720197- common product ion *m/z* 130.0652

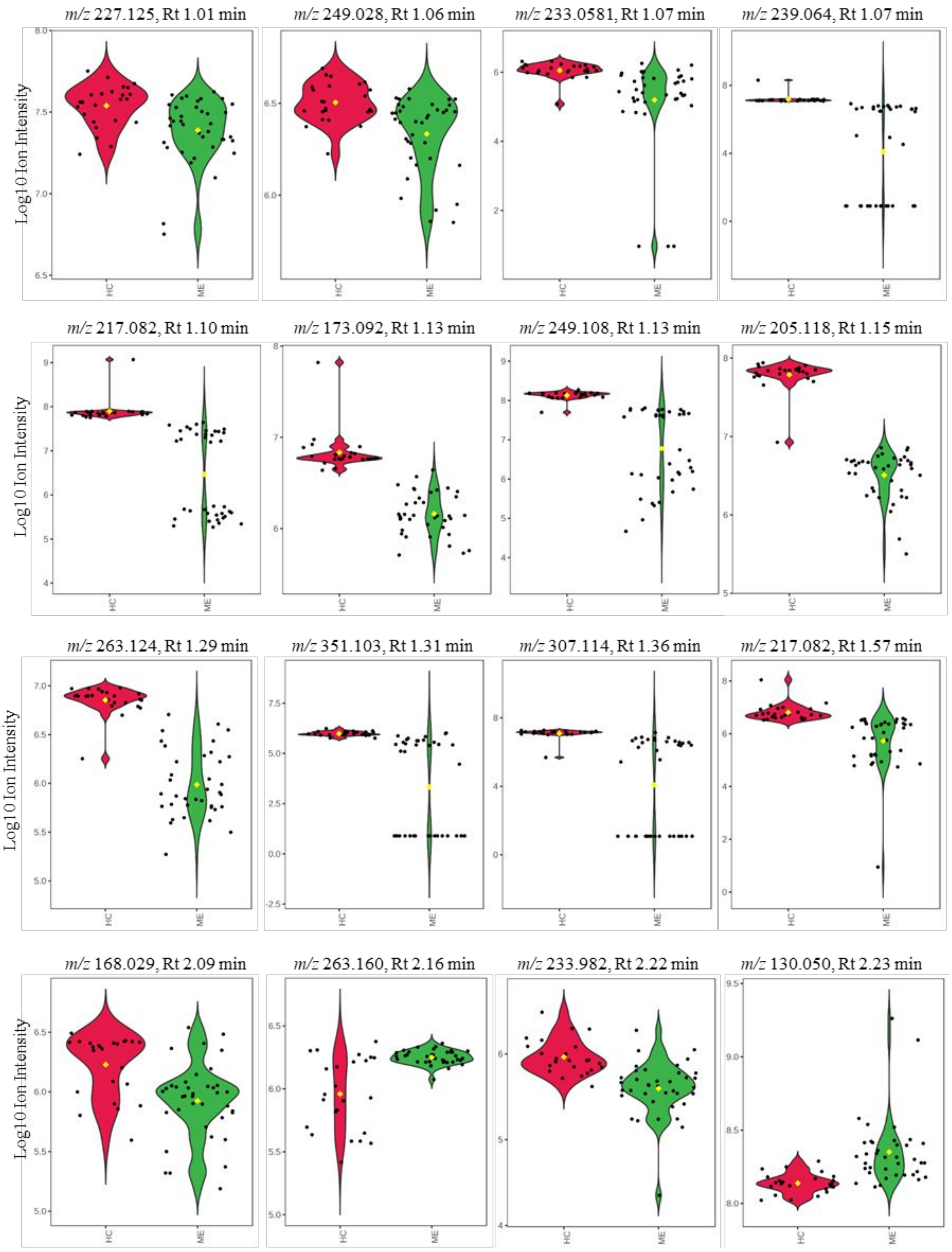
m/z 227.125, Rt 1.01 min
m/z 249.028, Rt 1.06 min
m/z 233.0581, Rt 1.07 min
m/z 239.064, Rt 1.07 min
m/z 217.082, Rt 1.10 min
m/z 173.092, Rt 1.13 min
m/z 249.108, Rt 1.13 min
m/z 205.118, Rt 1.15 min
m/z 263.124, Rt 1.29 min
m/z 351.103, Rt 1.31 min
m/z 307.114, Rt 1.36 min
m/z 217.082, Rt 1.57 min
m/z 168.029, Rt 2.09 min
m/z 263.160, Rt 2.16 min
m/z 233.982, Rt 2.22 min
m/z 130.050, Rt 2.23 min
Log10 Ion Intensity
HC
ME

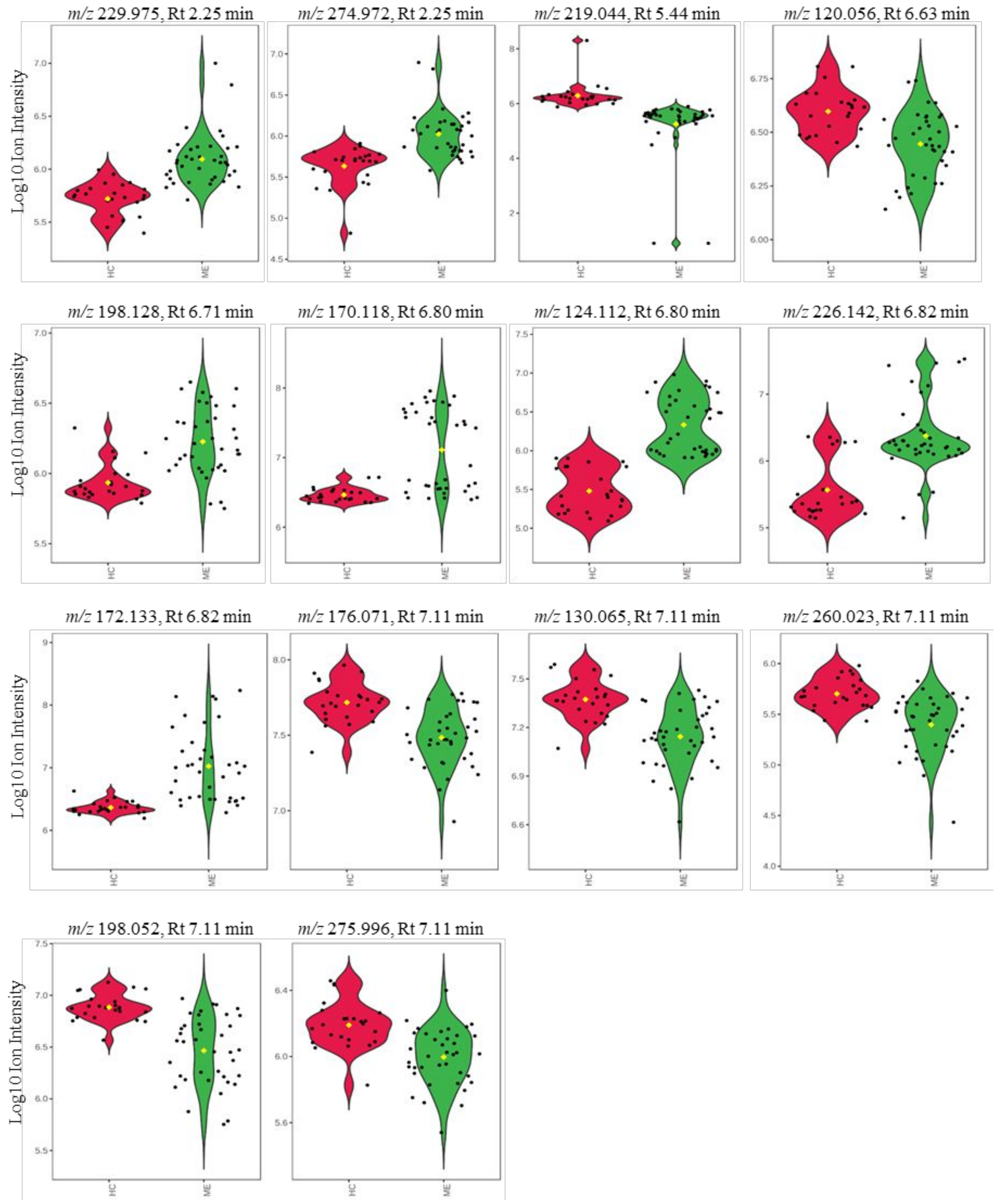

Figure S4. Violin plots depicting the distribution of the significant features between healthy control (HC) individuals (red color) and ME patients (green color). The ME group features were derived using the common features of the GC and SK cohorts. Each subplot corresponds to a different feature labelled with mass and retention time, showing the distribution density along with individual data points. The horizontal axis labels the group (HC or ME), while the vertical axis represents the log-transformed intensity of the features.

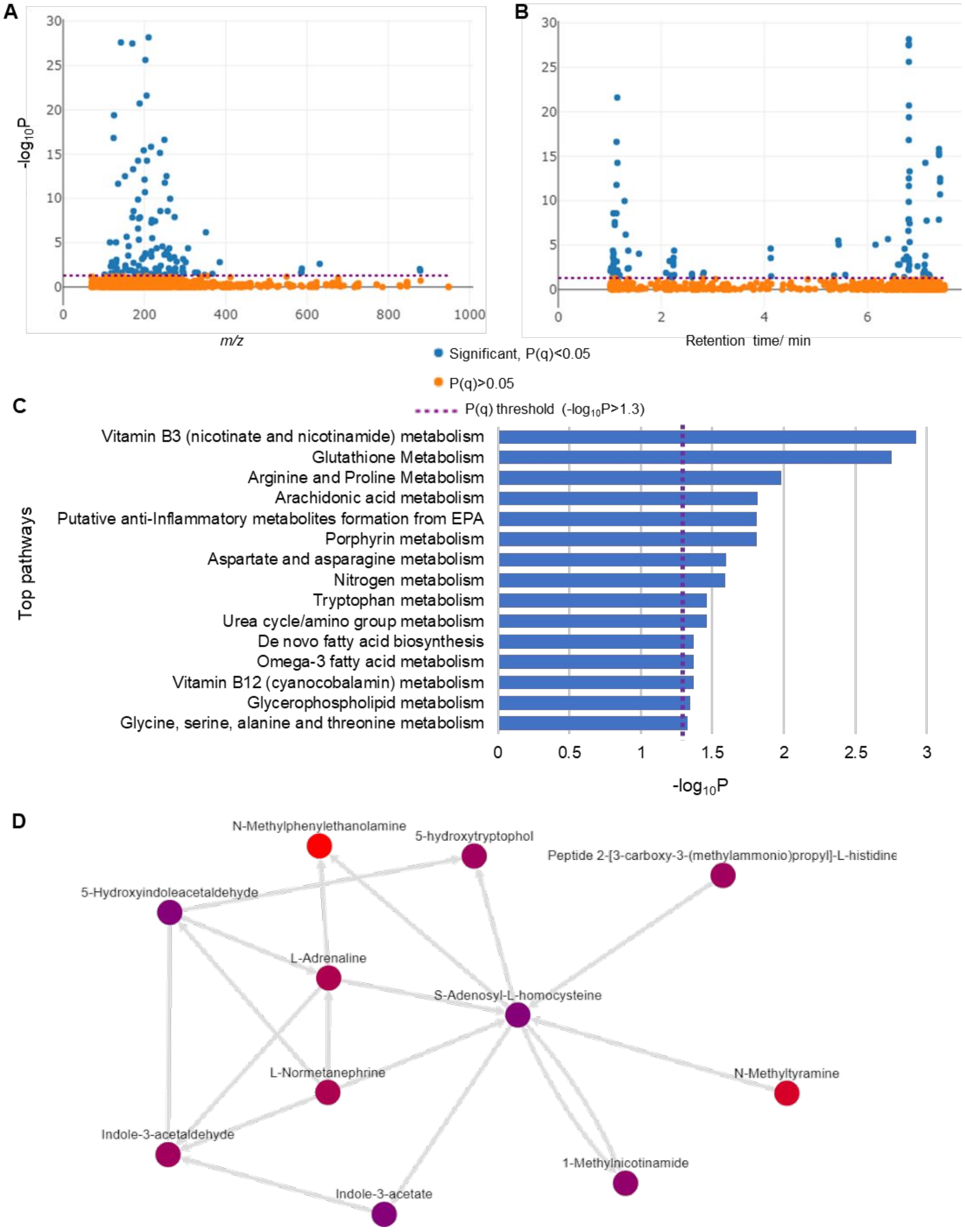

Figure S5. Untargeted metabolic pathway analysis between HC and ME-GC. A, B) Manhattan plots of the significance (-log10P) with the *m/z* and the retention time, respectively. C) Top pathways detected to be significant between HC and ME-GC. D) Activity network combining the results of pathway/module analyses.

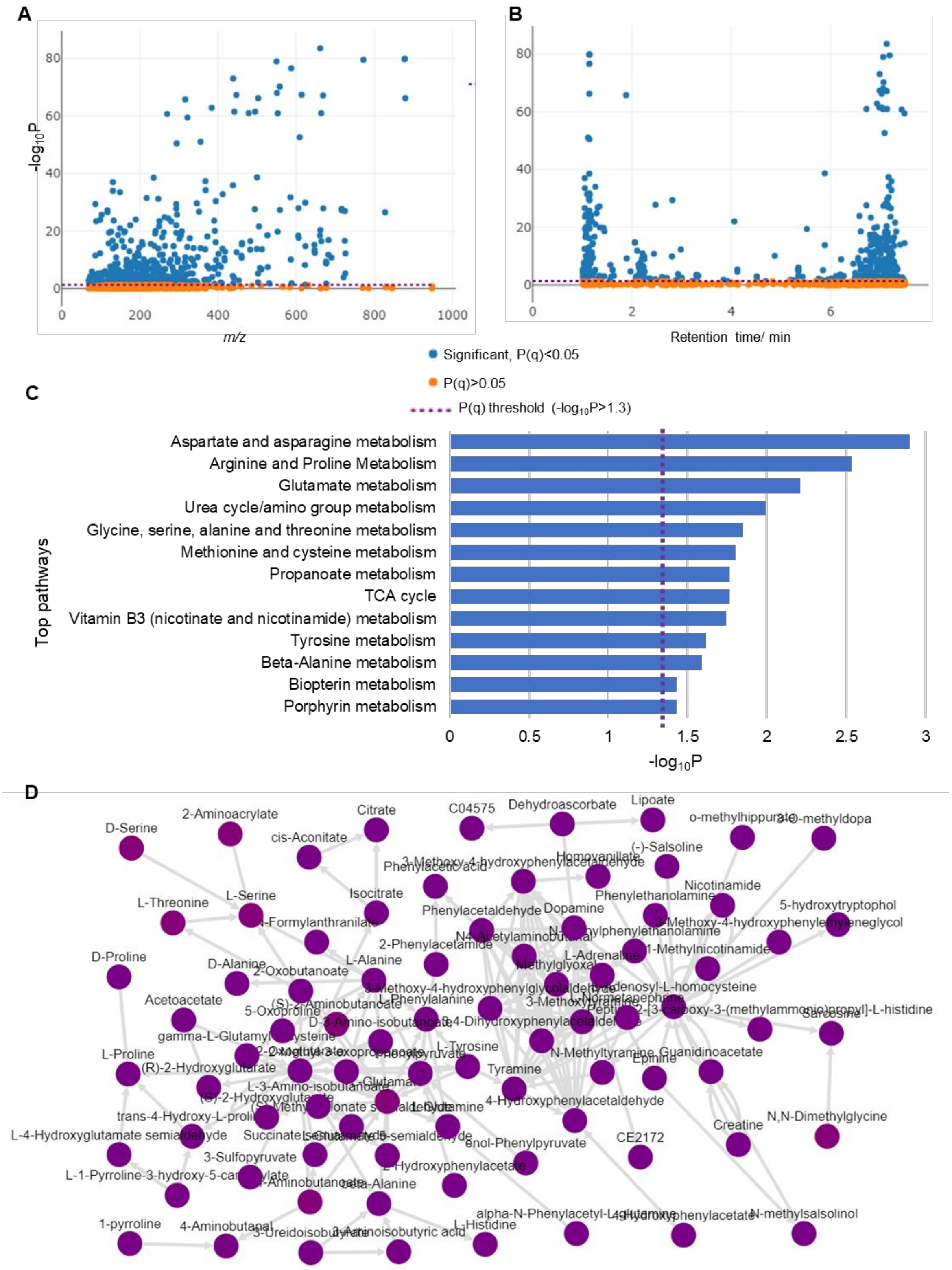

Figure S6. Untargeted metabolic pathway analysis between HC and ME-SK. A, B) Manhattan plots of the significance (-log10P) with the *m/z* and the retention time, respectively. C) Top pathways detected to be significant between HC and ME-SK. D) Activity network combining the results of pathway/module analyses.

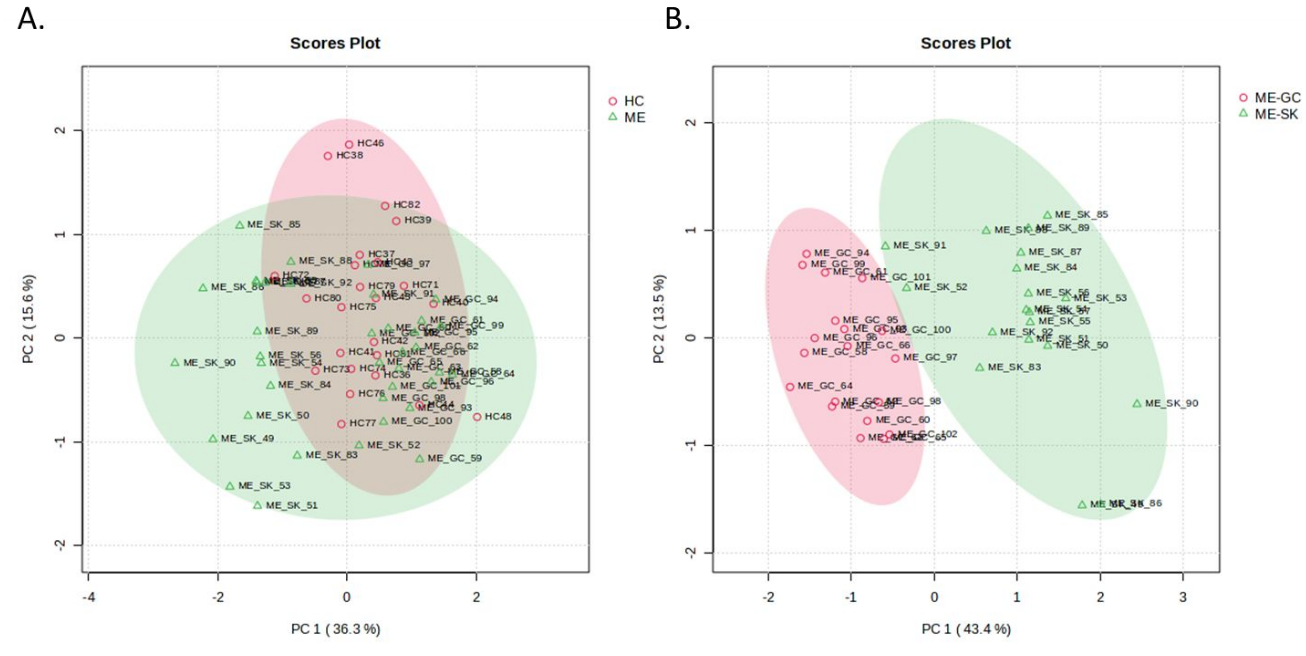

Figure S7. PCA plots are presented to show the distribution of HC, and ME samples for the targeted method. Panel (A) illustrates the variance between HC versus ME. (B) highlights that the Stockholm (SK) cohort of the ME/CFS patients is distinct from the Gothenburg (GC) cohort.

Table S2. The linear model with covariate adjustments was selected to assess the significance of sex on analyte concentration, with cohort type (HC vs. ME) and age included as covariates to control for their effects. Additionally, the Pearson correlation coefficient (Pearson r) was chosen to assess the correlation between analytes and age using cohort type (HC vs. ME) and sex as covariates.

Analyte	Sex covariate (p-value)	Age Correlation (Pearson r)	Age (p-value)
**Kynurenine**	**0.001**	0.48	**0.0001**
**Kynurenic acid**	**0.003**	0.19	0.14
**Quinolinic acid**	**0.046**	0.42	**0.001**
**Hydroxyanthranilic acid**	0.059	0.29	**0.02**
Tyrosine	0.097	0.17	0.19
Tryptophan	0.108	-0.14	0.28
Hypoxanthine	0.443	-0.03	0.84
Pantothenic acid	0.446	-0.01	0.94
**Hydroxykynurenine**	0.547	0.43	**0.001**
Serotonin	0.600	0.11	0.41
Riboflavin	0.694	-0.01	0.97
Nicotinamide	0.765	-0.08	0.57
Phenylalanine	0.927	-0.03	0.81

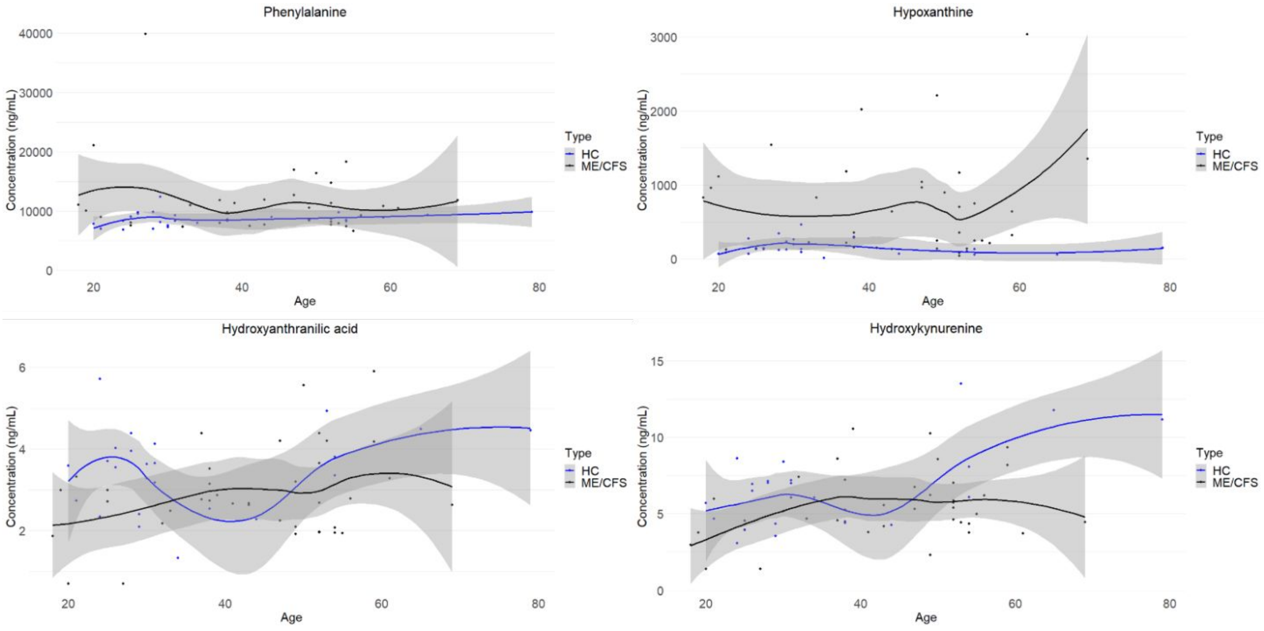

Figure S8. The scatter plots of the analytes that were significantly different between healthy controls (HC) and myalgic encephalomyelitis/chronic fatigue syndrome (ME/CFS), show the relationship between analyte concentration and age, categorized by health status. Each plot represents a specific analyte, with age on the x-axis and analyte concentration on the y-axis. Each participant is a data point, color-coded by HC or ME/CFS. A smooth curve illustrates the age-concentration trend. A grey shaded area, represents the confidence interval. These plots provide insight into analyte concentration variations across age groups and between ME/CFS and healthy controls.

Table S3. Plasma levels of tryptophan metabolites ratios in healthy control (HC) and myalgic encephalomyelitis/chronic fatigue syndrome (ME/CFS) patients.

Ratio	Sex	HC Median	ME/CFS Median	HC vs ME/CFS p-value
3HAA/QA	Female	0.06	0.05	0.17
	Male	0.07	0.05	
3HK/3HAA	Female	1.87	1.96	0.45
	Male	1.78	1.61	
**Kyn/3HK**	Female	50.32	59.92	0.02
	Male	53.98	77.33	
Kyn/KA	Female	40.35	46.15	0.15
	Male	41.09	42.49	
Phe/Tyr	Female	0.76	0.78	0.11
	Male	0.67	0.72	
Trp/Kyn	Female	40.85	35.06	0.23
	Male	30.87	34.47	
**Trp/5-HT**	Female	776.33	742.69	0.01
	Male	758.00	5166.16	

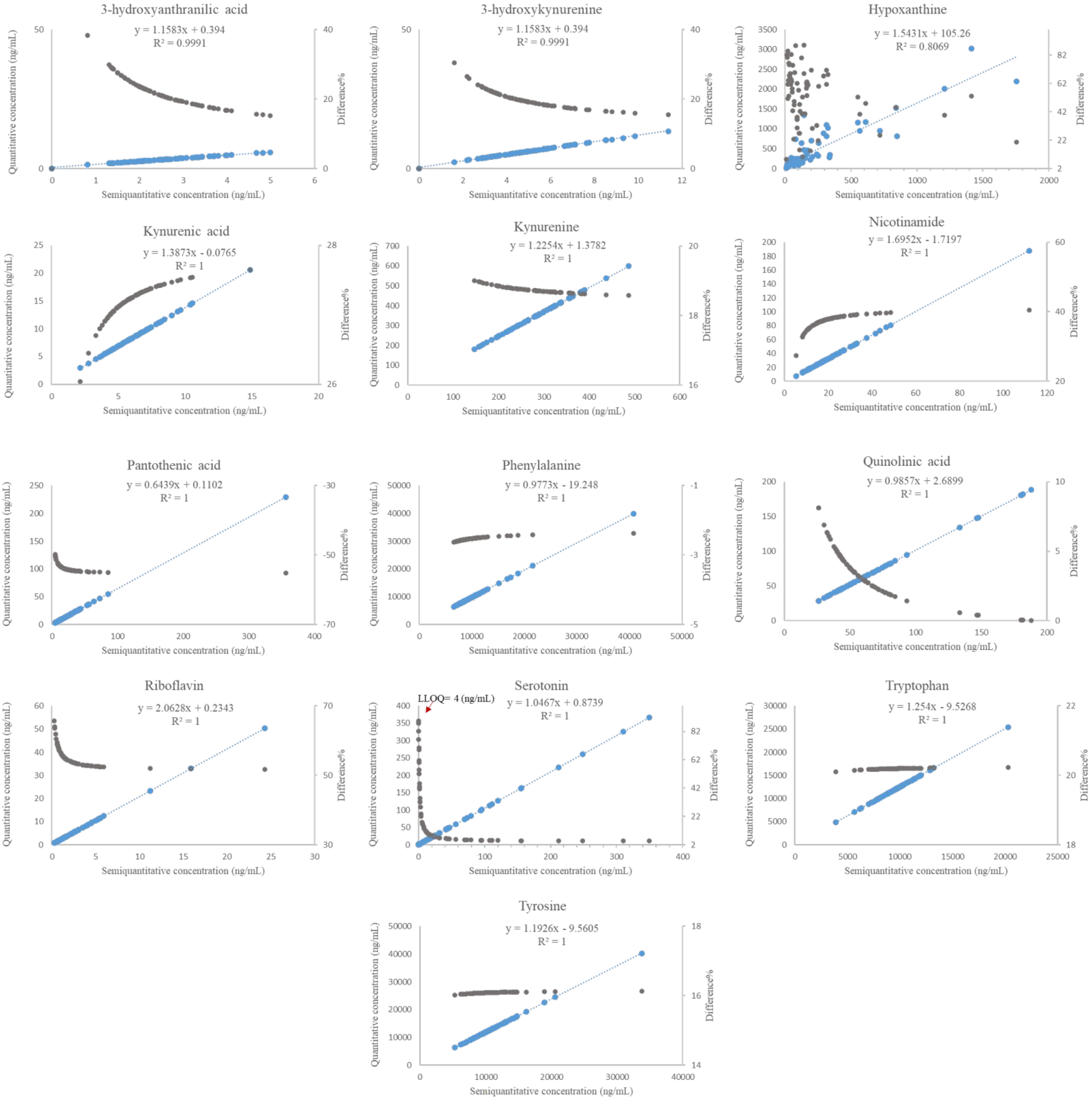

Figure S9. Comparison of tryptophan metabolites, phenylalanine, tyrosine, riboflavin, pantothenic acid, and hypoxanthine between two methods: quantitative analysis using an eight-point calibration curve and semi quantitative analysis using, the area ratio of the analyte to a relative internal standard, multiplied by the concentration of the internal standard. This comparison was conducted for plasma from myalgic encephalomyelitis (ME/CFS) patients and healthy controls. The results indicate a strong correlation (blue line) between the quantitative and semiquantitative results, with minimal differences observed for most compounds (grey line with the y-axis labelled Difference %). However, for certain analytes such as hypoxanthine, quinolinic acid-[$^{13}C_4$,^{15}N] was used as the internal standard; for pantothenic acid, theobromine was employed as the internal standard; and for

riboflavin, biotin-[$^2H_2$] was used as the internal standard, which might lead to a higher deviation in the results. Additionally, it is notable that deviations were higher at lower concentrations and the deviation declined with increasing concentration levels.
